# Associations Between Emotional Resilience and Mental Health Among Chinese Adolescents in the School Context: The Mediating Role of Positive Emotions

**DOI:** 10.3390/bs15050567

**Published:** 2025-04-23

**Authors:** Zhongmin Zhu, Biao Sang, Junsheng Liu, Yuyang Zhao, Ying Liu

**Affiliations:** 1 Shanghai Academy of Educational Sciences, Shanghai 200032, China; zzmin@cnsaes.org.cn; 2 Lab for Educational Big Data and Policymaking, Ministry of Education, P.R. China, Shanghai 200032, China; 3 School of Psychology and Cognitive Science, East China Normal University, Shanghai 200062, China; jsliu@psy.ecnu.edu.cn; 4 Department of Social Work, School of Sociology and Political Science, Shanghai University, Shanghai 200444, China; yuyang_zhao@yahoo.com; 5 Wenbo College, East China University of Political Science and Law, Shanghai 200042, China; liuying3729@126.com

**Keywords:** adolescents, emotional resilience, positive emotions, mental health

## Abstract

Positive emotions play an essential role in adolescent resilience and mental healthy development, yet whether it affects emotional resilience, mental health, and the internal mechanism remains unknown. Therefore, the current study aims to, using a two-wave panel design, examine the relationship between emotional resilience and mental health, as well as the mediating role of positive emotion. We conducted this longitudinal study in two waves with a 6-month interval, surveyed 266 Chinese adolescents (54.9% boys, *M*_age_ = 14.11 years, *SD* = 1.77), and constructed a mediation model. The participants completed the measures of demographic information, positive emotions, emotional resilience, and mental health at two times. The results revealed that after controlling for gender and age, Time 2 positive emotions partially mediated the relationship between Time 1 emotional resilience and Time 2 mental health. In detail, emotional resilience is positively correlated with life satisfaction and self-esteem. It shows a negative correlation with symptoms of depression and anxiety, partly mediated by positive emotions. The findings highlighted the role of emotional resilience in mitigating psychological problems and enhancing mental health in Chinese adolescents. The implications and limitations were discussed.

## 1. Introduction

Adolescence is a particularly crucial period for emotional development, as it presents opportunities for personal growth and the cultivation of lifelong well-being, as well as associated with a significant risk for mental health (Anderson & Priebe, 2021). During this period, episodes of mental health issues are common, with 10% to 20% of adolescents globally affected by mental health problems (Aguirre Velasco et al., 2020; Ma et al., 2021). It is crucial and urgent to comprehensively understand the factors and mechanisms that protect and harm adolescent mental health. Existing cross-sectional evidences demonstrated that emotional resilience as a type of resilience is recognized as an essential protective factor for adolescent mental health (i.e., Shapero et al., 2019; Wu et al., 2020).

Emotional resilience, stemming from the term “psychological resilience”, is characterized by the ability of an individual to improve or maintain positive emotions and recover from negative emotions when faced with stress and adversity (Davidson, 2000). Previous documents have suggested that the emotional resilience linked with depression and anxiety symptoms (Davis, 2009; Mesman et al., 2021; Shi et al., 2022), well-being (Sterina et al., 2022), and self-efficacy (Rudolph et al., 2025). Moreover, existing findings have shown that emotional resilience is associated with positive emotion (Gloria & Steinhardt, 2016) and that positive emotion is crucial to mental health (Alexander et al., 2021). However, there is no research examining the positive emotion function as a mediational role of emotional resilience and mental health. Moreover, previous studies addressing the associations between emotional resilience and mental health are mostly cross-sectional designs, while data analysis methods focus on correlation and regression analysis rather than exploring causational relationships (i.e., Gatt et al., 2020; Li et al., 2020; Wu et al., 2020). To address abovementioned research gaps, the present study aimed to examine emotional resilience and positive emotion and explore potential mediating mechanisms for enhancing adolescent mental health through a two-wave panel design. The current study contributes to a deeper understanding of the positive emotional pathways that link emotional resilience and mental health, thereby broadening the existing body of research in this field. Furthermore, it provides valuable implications for the development of interventions aimed at promoting adolescent mental health.

### 1.1. Emotional Resilience and Mental Health

According to the Complete Mental Health Theory put forward by Keyes (2007), complete mental health implies not only the absence of disease but also a positive and flourishing mental state, which can lead to a high level of emotional and social adjustment (Arslan & Allen, 2022; Arslan & Renshaw, 2018). Given the theoretical points, complete mental health was supposed to contain two aspects: positive mental health and negative mental health (Mesman et al., 2021). Draper et al. (2022) identified depression and anxiety as indicators of negative mental health, while life satisfaction and self-esteem were also included in the indicators of positive mental health in previous studies (Moksnes & Espnes, 2013; Zhou & Cheng, 2022). Consequently, we chose positive mental health indicators (i.e., life satisfaction, self-esteem) and negative mental health indicators (i.e., depression, anxiety).

Previous research showed that there was a close and significant relationship between emotional resilience and negative mental health indicators such as depression and anxiety among adolescents (Chung et al., 2020; Ramos-Díaz et al., 2019). For example, Chung et al. (2020) found that adolescents who lived with single parents showed lower emotional resilience, which in turn was related to high levels of depression. Also, the results of a study conducted by Ramos-Díaz et al. (2019) highlighted that the importance of developing resilience improves life satisfaction among adolescents. High levels of emotional resilience trigger multiple and flexible coping strategies among adolescents as a result, which could generate high levels of self-esteem by solving problems easily (Chung et al., 2020). In spite of this, the possible mediated variable has not been examined. From the perspective of positive psychology, positive emotion is a vital variable that has received more attention in recent years.

### 1.2. Emotional Resilience and Positive Emotions

Positive emotions are featured by the pleasure feelings that arise when individuals’ physical and mental needs are satisfied (Fredrickson, 2009). It is important to note that positive emotion and emotional resilience are both theoretically and functionally distinct constructs. Emotional resilience emphasizes a dynamic capacity to recover from adversity, rooted in stress-coping models that underscore cognitive–behavioral regulation (e.g., threat appraisal; Lazarus & Folkman, 1984; Ward et al., 2021). It prioritizes adaptive processes (e.g., restoring equilibrium) rather than transient affective states. In contrast, positive emotion emphasizes short-term hedonic experiences (e.g., joy) that enhance cognitive flexibility, as articulated in Fredrickson’s (1998) broaden-and-build theory. While positive emotions may temporarily alleviate negative emotions states (Fredrickson et al., 2000), they lack the sustained self-regulatory mechanisms that are central to resilience. Crucially, emotional resilience involves actively confronting adversity (e.g., tolerating uncertainty), whereas positive emotions can arise independently of adversity (Tugade & Fredrickson, 2004). Thus, emotional resilience represents a capacity for recovery, while positive emotion constitutes an affective outcome that may coexist with, but is not essential to, resilient processes.

The broaden-and-build theory (BBT) supposed by Fredrickson (1998) of positive emotions highlights that if the relationship between emotional resilience and positive emotion is reciprocal, positive emotion can build emotional resilience, and the build of emotional resilience can generate more positive emotion (Fredrickson, 1998). It is worth noting that previous studies have focused more on the influence of positive emotion on emotional resilience (i.e., Gloria & Steinhardt, 2016; Zhu et al., 2023). However, an increasing amount of evidence regarding emotional resilience has shown strong associations with positive emotions recently (Gilchrist et al., 2023). For instance, a study involving adolescents demonstrated that those with low emotional resilience exhibited lower levels of emotional pleasure compared to their counterparts with high emotional resilience (Cohn et al., 2009). In another study involving 421 college students who reported their mood states, the results from implementing a structural equation model indicated that one dimension of emotional resilience, which is the capacity to generate positive emotions, could foster the emergence of positive emotions and contribute to the attenuation of negative emotions. Meanwhile, another dimension of emotional resilience, which is the ability to recover from negative emotions, also directly lessened negative emotions (Y. Wang et al., 2016). Overall, emotional resilience has been recognized as to be associated with positive emotions, whereas how emotional resilience predicts positive emotions needs to be further examined.

### 1.3. Positive Emotion and Mental Health

In the field of mental health, researchers have argued that positive emotions are crucial for complete mental health (Keyes, 2007). Recently, an increasing number of researchers have differentiated positive emotion and mental health as two distinct constructs through various operational definitions and different measurements (Fredrickson & Joiner, 2018; Z. Wang et al., 2011). Positive emotions as emotional states that are related to the satisfaction of personal needs and are usually accompanied by the subjective experience of pleasure, including both transient emotions (i.e., pleasure) and diffuse and persistent positive emotions (Z. Wang et al., 2011). Mental health is a functional component of an individual’s overall psychological quality (L. Chen & Zhang, 2009). Therefore, the scope of mental health is larger and relatively stable than positive emotions and is generally used as an outcome variable in studies.

The undoing hypothesis proposed that positive emotions facilitate recovery from the autonomic arousal associated with negative emotions (Fredrickson & Levenson, 1998), indicating that positive emotions impact on negative indicators of mental health in a positive way. Furthermore, an individual with positive emotions in daily life or work experiences a high level of self-esteem (Nezlek & Kuppens, 2008), satisfies their own basic psychological and physical needs (Schutte & Malouff, 2021), and has access to complete mental health easily (Dong et al., 2012; Fredrickson & Joiner, 2018). Correspondingly, positive emotions helped individuals to relieve depression, fear, and anxiety (Santos et al., 2013). Cultivating positive emotions was an effective way to eliminate individual psychological barriers and promote healthy mental status (Gloria & Steinhardt, 2016). However, how to improve mental health through positive emotion remains to be addressed.

### 1.4. The Present Study

To address these gaps, the present study utilizes a two-wave panel design to investigate how emotional resilience predicts mental health outcomes among adolescents, and whether positive emotions serve as a key mediating mechanism in this relationship. By incorporating both positive and negative indicators of mental health, this study offers a more balanced and developmentally sensitive understanding of psychological well-being during adolescence. Given the existing evidence and the aims of the current study, the hypotheses were supposed as follows:

**H1:**  *Emotional resilience significantly predicts mental health among Chinese adolescents.*

**H2:**  *Positive emotions mediate the relationship between emotional resilience and mental health.*

**H3:**  *T2 positive emotions mediate the relationship between T1 emotional resilience and T2 mental health.*

## 2. Methods

### 2.1. Participants

A longitudinal study was conducted involving three high school classes and five middle school classes. These medium-sized schools are located in urban areas of moderate socio-economic status. A total of 291 questionnaires were distributed, resulting in a final sample of 266 adolescents, comprising 146 boys (54.9%) and 120 girls (45.1%). The participants’ ages ranged from 12 to 18 years, with a mean age of 14.11 years (*SD* = 1.77). Over 90% of the students were of Han nationality, the predominant ethnic group in China.

### 2.2. Measures

Emotional resilience was measured using the Adolescents’ Emotional Resilience Questionnaire (AERQ) developed by Zhang and Lu (2010). The AERQ contains 11 items that yield two dimensions: the ability to generate positive emotions (GPE) (e.g., ‘When I’m in a bad mood, I can think of happy things’) and the ability to recover from negative emotions (RNE) (e.g., ‘I can adjust my negative emotions in a short time’). Each item is rated on a 6-point scale (from 1 = completely disagree to 6 = completely agree). A higher score indicates a greater level of emotional resilience. During the T1 assessment, the overall scale demonstrated good reliability, with Cronbach’s α = 0.91. Furthermore, the reliabilities for the GPE and RNE dimensions were also very good at 0.90 and 0.85, respectively.

Positive emotion was measured using the positive affect items from the Positive Affect and Negative Affect Scale for Children (PANAS-C) (Pan et al., 2015), originally developed by Laurent et al. (1999). This measure consists of 15 items; each item describes a positive emotion, such as ‘happy’, among others. Participants rated their own emotional levels over recent weeks using a 5-point scale ranging from 1 (none) to 5 (very much). Higher total scores reflect higher levels of positive emotion. In this study, Cronbach’s α for the Positive Emotion Questionnaire was found to be 0.92.

Life satisfaction was evaluated using the Satisfaction with Life Scale (SWLS), which includes five items and was developed by Diener et al. (1985). This scale has been widely utilized in China with established reliability and validity (Jiang et al., 2018). Participants rated their perceptions of life quality on a 7-point scale ranging from 1 (strongly disagree) to 7 (strongly agree), responding to statements such as ‘I am satisfied with my life’. Higher total scores indicate greater life satisfaction; in this study, Cronbach’s α for this measure was reported at 0.83.

Self-esteem was measured using the 10-item Self-Esteem Scale (Wu et al., 2017), which uses 5 scoring points ranging from 1 (complete non-conformity) to 5 (complete conformity). A higher total score indicated a higher level of self-esteem (e.g., ‘Overall, I am satisfied with myself’). Previous studies have shown that the scale has good reliability and validity (Wu et al., 2017; Xie et al., 2019). Cronbach’s α in this study was 0.87.

Depression was assessed using the Center for Epidemic Studies Depression Scale (CES-D), which comprises 20 items (Radloff, 1977). This scale required participants to subjectively evaluate the frequency of depressive symptoms experienced over the past week (e.g., ‘It’s hard for me to concentrate’), and it has been previously validated in Chinese adolescents (Tang et al., 2019). The questionnaire employed a 4-point scale ranging from 0 to 3, where scores of 0, 1, 2, and 3 corresponded to occasional or none (less than one day per week), sometimes (1–2 days per week), frequently or about half the time (3–4 days per week), and most of the time or nearly every day (5–7 days per week). A higher total score indicated a greater level of depressive symptoms. In this study, Cronbach’s α was found to be 0.91.

Anxiety was assessed using the ‘Trait Anxiety Questionnaire’, which consists of 20 items adapted from prior research (J. Chen et al., 2015; Shek, 1993). Participants were instructed to reflect on their own emotional experiences and provide ratings for four statements presented in the scale (e.g., ‘I worry too much about things that don’t really matter’), with a scoring system ranging from 1 (almost none) to 4 (almost always). A higher total score indicated a greater level of anxiety. Previous studies have demonstrated that the reliability and validity of this questionnaire fulfill psychometric standards (J. Chen et al., 2015; Shek, 1993). In this study, Cronbach’s α was found to be 0.89.

### 2.3. Procedures

The current study received initial approval from the Institutional Review Board (IRB) of BLINDED University prior to data collection. Data for Time 1 (T1) were collected in April and May of 2021. Subsequently, all Time 2 (T2) data were gathered six months after T1, specifically in October and November of 2021. Participants included students aged 12 to 18, as well as their parents, who were informed about this study and asked to provide voluntary consent by signing an informed consent form. Students had the right to withdraw from this research at any time, even if they had previously signed the informed consent.

All data utilized in this study were obtained from students through a paper-based questionnaire. Trained investigators administered these questionnaires by providing face-to-face instructions to all participating students. Afterward, the investigators collected the completed questionnaires and securely stored them in a laboratory setting for further analysis.

### 2.4. Statistical Analysis

The analyses conducted in the current study utilized SPSS 22.0 and the SPSS Process Macro. First, we performed a test for common method bias to confirm that there was no significant effect of common method bias present. Second, we examined the correlations among the variables under investigation. Finally, we employed the SPSS Process Macro to assess the mediating role of positive emotions between emotional resilience and mental health, utilizing bias-corrected bootstrap tests with a 95% confidence interval to determine whether the indirect effects were statistically significant.

## 3. Results

### 3.1. The Control and Verification of Common Method Variance

Since all variables utilized in the current study were measured through students’ self-reports, there is a potential for common method variance (CMV) effects. To assess this possibility, the Harman single-factor test was conducted to evaluate CMV (Podsakoff et al., 2003). All variables were subjected to exploratory factor analysis without rotation.

For the T1 assessment, results indicated that there were 13 factors with characteristic roots greater than 1, and the variance explained by the first factor was 31.88%, which falls below the critical threshold of 40%. In the T2 assessment, findings revealed that there were 15 factors with characteristic roots exceeding 1, and the variance accounted for by the first factor was 32.73%, also less than the critical value of 40%. Consequently, no significant CMV effects were identified in this study.

### 3.2. Descriptive Statistics

As presented in Table 1, the descriptive statistics and the interrelationships among T1 emotional resilience, T2 positive emotions, and T2 mental health indicators are reported. The findings revealed a robust positive correlation between T1 emotional resilience, T2 positive emotions, and the positive indicators of mental health at T2 (i.e., life satisfaction and self-esteem). In contrast, there exists a significant negative correlation between T1 emotional resilience, T2 positive emotions, and the negative indicators of mental health at T2 (i.e., depression and anxiety).

### 3.3. The Mediating Effect of Positive Emotion on the Relationship Between Emotional Resilience and Mental Health

**Figure 1:**
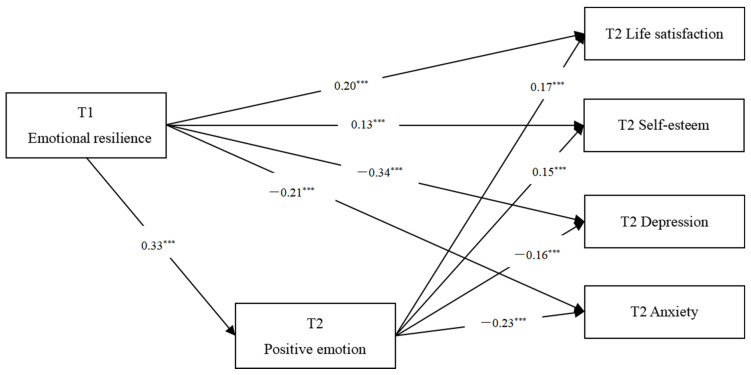
The mediating model of positive emotion on the relationship between emotional resilience and mental health. Note. *** *p* < 0.001.

By using the SPSS Process Macro, the mediating effects of T2 positive emotions on the relationship between T1 emotional resilience and four T2 mental health indicators were tested. As shown in Table 2, after controlling for gender and age, the results suggested that T2 positive emotions partially mediated the relationship between T1 emotional resilience and T2 mental health. The Bootstrap test results showed that the 95% confidence interval did not include the number 0, and the mediating effect was significant. As can be seen from Figure 1, the total and direct effects of emotional resilience were also significant.

## 4. Discussion

This study explored the impact of emotional resilience on adolescent mental health in a two-wave panel design, highlighting the mediating role of positive emotion. The findings indicated the critical role emotional resilience plays in both reducing mental health problems and promoting positive mental health outcomes. Specifically, the results demonstrate that emotional resilience positively influences life satisfaction and self-esteem while mitigating the effect of depression and anxiety. Moreover, positive emotions partially mediate these relationships, indicating that emotional resilience not only exerts direct effects on mental health but also facilitates improvements through the enhancement of positive emotional experiences.

Previous studies found that psychological resilience had a significant influence on mental health (Liu et al., 2019; Chung et al., 2020; Gloria & Steinhardt, 2016). The current study focused on a specific type of psychological resilience, emotional resilience, and found that it also had a significant effect on mental health, consistent with H1, which deepens the research on the relationship between psychological resilience and mental health. The integration of positive and negative mental health indicators into a single framework aligns with Keyes’ theory of complete mental health (Keyes, 2007). This approach underscores the importance of addressing both the reduction of mental illnesses and the promotion of flourishing mental states. By focusing on a sample of adolescents, this study expands the literature beyond younger adults and seniors, offering valuable insights into a critical developmental period. Moreover, the broaden-and-build theory of positive emotions (Fredrickson, 1998) provides a theoretical basis for understanding the mechanisms through which emotional resilience fosters mental health. Positive emotions, as posited by this theory, not only broaden cognitive and behavioral repertoires but also build enduring psychological resources such as resilience and well-being. The current findings align with prior research, suggesting that positive emotions serve as a key driver in this dynamic process.

The significant mediating effect of positive emotions indicated that they played a significant role in promoting mental health, consistent with H2 and H3. Positive emotions have been a cornerstone of contemporary positive psychology, receiving considerable attention for their role in enhancing mental health. Seligman (2018) considered positive emotions as one of the three main concepts of positive psychology and built the PERMA model of positive psychology. Seligmans’s PERMA model underscores the importance of positive emotions as one of five key pillars of well-being, alongside engagement, relationships, meaning, and achievement. Positive emotions not only initiate and sustain positive mental states but also counterbalance the human tendency to focus on negative experiences. Extensive research on positive emotions emerged during the beginning of the 20th century; more attention was given to the important role of positive emotions on mental health (Gruber & Purcell, 2015; Sang et al., 2014). Meanwhile, positive emotions are usually associated with positive feedback on individuals’ behaviors. When individuals experience positive emotions while they are performing certain activities, they are more likely to show such behaviors, especially for healthy behavior. Positive emotions promote mental and physical health. People should cultivate positive emotions in their daily lives not only because they feel good in the moment but also because they help them to feel better and lead them to prosperity, health, and longevity. The BBT of positive emotions emphasized that positive emotions were the core factors in helping individuals reach their optimum functions and moving forward to a more psychologically healthy status (Fredrickson, 2009; Fredrickson & Joiner, 2018).

### 4.1. Educational Implications from the Current Investigation

Integrating the findings from previous research (focused on how positive emotions promote emotional resilience) and the current study (focused on how emotional resilience promotes positive emotions), this study highlights the potential of positive emotions to create a virtuous cycle in building psychological resources. Positive emotions foster emotional resilience, which, in turn, generates further positive emotions, leading to a dynamic upward spiral of mental health and well-being.

Positive emotions can be cultivated in daily life through accessible activities such as interacting with others, helping others, playing, and learning. These activities utilize abundant endogenous resources that remain largely untapped (Troy & Mauss, 2011). Positive emotions generated in such contexts can stimulate a process of spiral escalation: positive emotions build psychological resources, and these resources lead to more positive emotions, creating a self-reinforcing cycle (Fredrickson & Joiner, 2018). Psychological resilience, while often associated with positive emotions experienced during difficult situations, is not necessarily linked to a reduction in negative emotional arousal but contributes meaningfully to overall well-being.

These findings provide important implications for promoting mental health practices. Individuals can actively cultivate positive emotions through small, intentional actions, carving a pathway to improved health and well-being (LaBelle, 2023). Interventions should focus on embedding these practices into educational and community programs to foster resilience and mental health on a larger scale.

### 4.2. Limitations and Future Research

Even though this study provides significant insights into the interplay between emotional resilience and positive emotions, several implications and avenues for further research emerge. First, the findings rely primarily on self-reported data, which could introduce biases. While this study utilized longitudinal data, future research should incorporate mixed methods, including behavioral experiments and cognitive neuroscience approaches, to yield more convergent conclusions about the influence of emotional resilience on mental health.

Second, more nuanced studies are needed to explore the effects of emotional resilience on specific types of positive emotions. Research suggests that positive emotions can be categorized into high-approach emotions (e.g., enthusiasm, desire, excitement) and low-approach emotions (e.g., contentment, satisfaction, love, gratitude) (Gilbert, 2012). High-approach emotions are associated with reward-seeking behaviors and motivate actions, whereas low-approach emotions emphasize savoring the present moment without necessarily prompting action. Understanding whether emotional resilience exerts differential effects on these categories, and how each influences mental health, remains an important area for future research.

Third, while positive emotions generally enhance mental health, disturbances in positive emotional regulation may have adverse effects. Excessive positive emotions have been linked to clinical syndromes such as problematic drug and alcohol use, risky sexual behavior, bulimia, gambling, and mania (Gruber & Purcell, 2015). Individuals who fail to down-regulate overly heightened positive emotions are particularly susceptible to these risks. Future study could explore the boundaries of positive emotional experiences on different types of behaviors, as well as the strategies to regulated excessive positive emotions, which will provide theoretical evidence for psychological and educational interventions.

Lastly, the reliance on two-time-point data restricts the ability to model nonlinear trajectories, limiting insights into dynamic processes that may unfold over multiple intervals. Methodologically, while the SPSS Process Macro was selected for its accessibility and capacity to test mediation, future study might employee latent variable modeling or advanced longitudinal techniques (e.g., cross-lagged models) to replicate this study. Additionally, the absence of measurement invariance testing between time points might raise concerns about whether observed changes reflect true differences or measurement artifacts; the observational design and limited temporal resolution preclude definitive conclusions about causality. Future research should incorporate multi-wave designs, validate measures across time points, and employ robust longitudinal analyses to strengthen temporal and causal claims.

## 5. Conclusions

This study highlights the pivotal role of emotional resilience in promoting adolescent mental health, both directly and through the mediating influence of positive emotions using a two-wave panel design. Integrating the findings from prior research (focused on how positive emotions foster emotional resilience) and the current study (focused on how emotional resilience promotes positive emotions), the results suggest a self-reinforcing cycle between positive emotions and psychological resources. Positive emotions facilitate the development of emotional resilience, which, in turn, fosters further positive emotional experiences, creating a dynamic upward spiral of well-being. Positive emotions can be cultivated in daily life through activities such as social interactions, acts of kindness, play, and learning. These easily accessible endogenous resources provide a foundation for improving health and well-being. By leveraging this positive cycle, individuals can enhance their mental health and achieve flourishing states.

## Figures and Tables

**Table 1:** Mean, standard deviation (SD), and correlation coefficient of emotional resilience, positive emotion, and mental health (n = 266).

Variables	*M*	*SD*	1	2	3	4	5	6
1. T1 Emotional resilience	41.62	12.90	-					
2. T2 Positive emotions	47.49	10.27	0.34 ***	-				
3. T2 Life satisfaction	22.92	5.94	0.44 ***	0.43 ***	-			
4. T2 Self-esteem	28.67	4.33	0.43 ***	0.43 ***	0.47 ***	-		
5. T2 Depression	16.19	8.79	−0.47 ***	−0.37 ***	−0.60 ***	−0.72 ***	-	
6. T2 Anxiety	43.80	7.18	−0.43 ***	−0.47 ***	−0.57 ***	−0.71 ***	0.78 ***	-

Note. *** *p* < 0.001.

**Table 2:** Test of mediating effect of positive emotions on the relationship between emotional resilience and mental health (n = 266).

Dependent Variable		Gender	Age	T1 Emotional Resilience	T2 Positive Emotion	Indirect Effect (95% Confidence Interval)
T2 Positive emotion	B	−0.85	−1.46	0.33		
	t	−0.53	−3.49 **	5.51 ***		
	95%CI	[−4.03, 2.33]	[−2.28, −0.64]	[0.21, 0.45]		
T2 Life satisfaction	B	0.71	0.17	0.20	0.17	0.06 [0.03, 0.10]
	t	0.82	0.74	5.66 ***	4.37 ***	
	95%CI	[−0.99, 2.41]	[−0.28, 0.63]	[0.13, 0.27]	[0.09, 0.25]	
T2 Self-esteem	B	−0.58	0.49	0.13	0.15	0.05 [0.03, 0.08]
	t	−0.93	2.96 **	5.22 ***	5.34***	
	95%CI	[−1.80, 0.65]	[0.16, 0.82]	[0.08, 0.18]	[0.09, 0.21]	
T2 Depression	B	0.13	0.23	−0.34	−0.16	−0.05 [−0.10, −0.02]
	t	0.10	0.68	−6.71 ***	−2.80 ***	
	95%CI	[−2.40, 2.67]	[−0.44, 0.91]	[−0.44, −0.24]	[−0.28, −0.05]	
T2 Anxiety	B	0.68	0.23	−0.21	−0.23	−0.08 [−0.13, −0.04]
	t	0.66	0.84	−4.95 ***	−4.86 ***	
	95%CI	[−1.36, 2.74]	[−0.32, 0.79]	[−0.29, −0.13]	[−0.32, −0.14]	

Note. ** *p* < 0.01, *** *p* < 0.001.

## Data Availability

Data are unavailable due to privacy restrictions and ethical concerns.
